# Structural variability of CG-rich DNA 18-mers accommodating double T–T mismatches

**DOI:** 10.1107/S2059798320014151

**Published:** 2020-11-24

**Authors:** Petr Kolenko, Jakub Svoboda, Jiří Černý, Tatsiana Charnavets, Bohdan Schneider

**Affiliations:** a Faculty of Nuclear Sciences and Physical Engineering, Czech Technical University in Prague, Brehova 7, 11519 Prague 1, Czech Republic; b Institute of Biotechnology of the Czech Academy of Sciences, BIOCEV, Prumyslova 595, 252 50 Vestec, Czech Republic

**Keywords:** DNA structure, T–T mismatch, noncanonical base pairs, repetitive extragenic palindromes, REPs, crystal structure, CD spectra

## Abstract

Solution and crystal data are reported for DNA 18-mers with sequences related to those of bacterial noncoding single-stranded DNA segments called repetitive extragenic palindromes (REPs). Solution CD and melting data showed that the CG-rich, near-palindromic REPs from various bacterial species exhibit dynamic temperature-dependent and concentration-dependent equilibria, including architectures compatible with not only hairpins, which are expected to be biologically relevant, but also antiparallel duplexes and bimolecular tetraplexes. Three 18-mer oligonucleotides named Hpar-18 (PDB entry 6rou), Chom-18 (PDB entry 6ros) and its brominated variant Chom-18Br (PDB entry 6ror) crystallized as isomorphic right-handed A-like duplexes. The low-resolution crystal structures were solved with the help of experimental phases for Chom-18Br. The center of the duplexes is formed by two successive T–T noncanonical base pairs (mismatches). They do not deform the double-helical geometry. The presence of T–T mismatches prompted an analysis of the geometries of these and other noncanonical pairs in other DNA crystals in terms of their fit to the experimental electron densities (RSCC) and their geometric fit to the NtC (dinucleotide conformational) classes (https://dnatco.datmos.org/). Throughout this work, knowledge of the NtC classes was used to refine and validate the crystal structures, and to analyze the mismatches.

## Abstract 2

Two investigated DNA 18-mers indicate a dynamic equilibrium of conformations in solution and crystallize as duplexes with two consecutive T–T mismatches. Neither these mismatched nucleotides nor others found in the PDB exhibit unique structural features compared with Watson–Crick paired nucleotides.

## 1. Introduction   

DNA self-recognition and its ability to store genetic information is mainly driven by the formation of canonical Watson–Crick base pairs. However, noncanonical pairs, also termed mismatched pairs in some literature, may be more important in DNA structures than has generally been appreciated (Saini *et al.*, 2013 ▸; Kaushik *et al.*, 2016 ▸). Noncanonical pairs are essential for the stabilization of various folded DNA forms such as guanine or i-motif quadruplexes, adenine-zipper motifs, triplexes, folded DNAzymes, hairpin stems and cruciforms, which may all play roles in various biological processes. These folded DNA forms may influence the kinetics of some biological processes (Tateishi-Karimata & Sugimoto, 2020 ▸), enable homologous recombination (Masuda *et al.*, 2009 ▸) or cause mitochondrial diseases (Damas *et al.*, 2012 ▸; Oliveira *et al.*, 2013 ▸). A specific role is played by two G- or C-rich non­canonical architectures: G-quadruplexes and i-motifs. Historically, much attention has particularly been paid to G-quadruplexes. These structures are known to regulate DNA transcription (Ravichandran *et al.*, 2019 ▸) and have a causal connection to several human diseases (Maizels, 2015 ▸), including roles in regulating the processing of a range of noncoding RNAs and linking them to neurodegenerative diseases (Simone *et al.*, 2015 ▸). The complementary C-rich strands can undergo hairpin–i-motif equilibration upon a pH change (Cristofari *et al.*, 2019 ▸) and, owing to their stability, impede DNA replication or repair (Takahashi *et al.*, 2017 ▸).

We are interested in a specific class of CG-rich DNA sequences called repetitive extragenic palindromes (REPs). REPs are DNA segments of about 30 nucleotides in length that occur frequently in some bacterial species. Several REPs and their inversions, iREPs, encompass the gene for a specific transposase called RAYT (REP-Associated tYrosine transposase; Nunvar *et al.*, 2010 ▸). Some bacterial species contain hundreds of REP–RAYT–iREP clusters belonging to BIMEs (bacterial interspersed mosaic elements), but their role in bacterial processes and the molecular mechanism of their transposition are unclear (Dyda *et al.*, 2012 ▸). Hairpin conformations are considered to be biologically relevant for the recognition of REP by RAYT proteins, as revealed by the only known structure of a REP–RAYT complex (Messing *et al.*, 2012 ▸). A previous biophysical study in solution (Charnavets *et al.*, 2015 ▸) showed that REPs from various bacterial species can also adopt conformations other than hairpins. Such structural and conformational variability of the REP sequences would be essential in the genomic context in order to participate in interactions with RAYT variants. Moreover, the equilibria between several conformational species of the REP oligo­nucleotides represent a possibility for regulating the nuclease and transposase activities of RAYT. Therefore, the unknown mechanism of RAYT transposition makes the REP–RAYT system an attractive subject for biochemical and structural studies with an impact on understanding the mechanisms that maintain the integrity of bacterial genomes.

**Figure 1:**
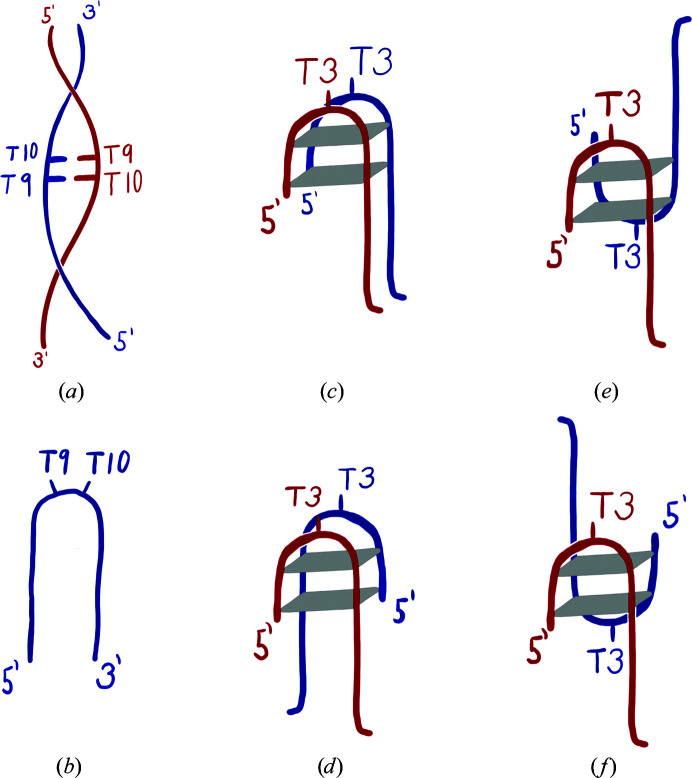
Possible topologies of the Hpar-18 and Chom-18 DNA oligonucleotides. Thymine residues forming loops or noncanonical base pairs are indicated.

In this study, we focus on two REP-related oligonucleotide sequences called Hpar-18 and Chom-18. We present their characterization in the liquid and crystal phases and then discuss in detail an important feature of the reported crystal structures: noncanonical base pairing. Both Hpar-18 and Chom-18 can acquire several molecular architectures, as outlined in Fig. 1 ▸, and our solution data confirm the previous observation (Charnavets *et al.*, 2015 ▸) that oligonucleotides with REP-related sequences adopt multiple conformations in dynamic temperature- and solution-dependent equilibria. In the crystal phase, these DNA 18-mers form double helices with two successive T–T mismatches in the center of the duplexes. These mismatches do not deform the duplex geometry. Therefore, we further analyzed the geometries of dinucleotides containing T–T and other mismatches in other crystal structures and observed that they mostly adopt the conformations known for Watson–Crick paired dinucleotides so that they do not disrupt the regular double-helical arrangement. The analysis of the mismatched segments from the database as well as the refinement of our crystal structures benefited from the knowledge of the nucleic acid dinucleotide (NtC) classes (Schneider *et al.*, 2018 ▸; Černý, Božíková, Svoboda *et al.*, 2020 ▸) and the tools available at the web server https://dnatco.datmos.org/ (Černý *et al.*, 2016 ▸), showing the potential of the NtC classification for an automated, strictly geometric analysis of nucleic acids.

## 2. Materials and methods   

### 2.1. Studied DNA oligonucleotides   

We studied two DNA 18-mers related to the REP sequences of the bacteria *Haemophilus parasuis* (Hpar-18) and *Cardiobacterium hominis* (Chom-18). The sequences retrieved from the bacterial genomes are available in the NCBI genomic repository. They are palindromic except for the central TT dinucleotide (highlighted in bold italics). The third oligo­nucleotide, Chom-18Br, is a brominated mutant of Chom-18. The names, sequences and PDB codes of the studied oligonucleotides are given below.
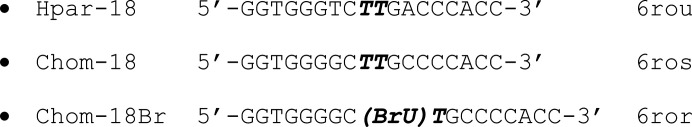


The oligonucleotides were purchased from Generi Biotech s.r.o. (Czech Republic). For the circular dichroism (CD) and absorbance measurements, the oligonucleotides were diluted to concentrations of 2 and 20 µ*M* in water, a pH 7.4 buffer containing 100 m*M* Na^+^ cations that was prepared by combining appropriate quantities of 59.8 m*M* NaCl, 20 m*M* Na_2_HPO_4_, 0.1 m*M* Na_2_EDTA and 79.8 m*M* NaCl, 20 m*M* NaH_2_PO_4_, 0.1 m*M* Na_2_EDTA, or crystal screen formulations. Prior to the experiments, the oligonucleotides were denatured by heating to 100°C for 5 min and cooled to room temperature. To explore the influence of strontium cations on the conformation of the Hpar-18 and Chom-18 oligonucleotides, strontium chloride at a 100 or 1000 m*M* stock concentration was added directly to the photometric cell and preheated to 100°C before measurement of the spectrum.

### 2.2. Circular-dichroism spectra and UV absorption thermal denaturation measurements   

CD spectroscopy was used to investigate the conformation of the oligonucleotides in solution. The spectra were recorded as a function of temperature using a Chirascan-plus spectrophotometer (Applied Photophysics, Leatherhead, UK) in steps of 1 nm over the wavelength range 205–340 nm with an averaging time of 1 s per step. Samples at a concentration of 20 µ*M* in 1 mm path-length quartz cells were placed into a thermostated cell holder and spectra were recorded at intervals of 5°C. The CD signal was obtained as ellipticity in units of millidegrees and the resulting spectra, after buffer-spectrum subtraction, were normalized by oligonucleotide concentration to yield molar ellipticities.

To ascertain the number of DNA conformers required to account for the observed spectral changes, we subjected the temperature-dependent CD spectra to single-value decomposition (SVD) using the *Global* 3 software. Any number greater than two indicates the presence of more than one conformation in the native state or the existence of intermediate species in the order–disorder transition.

Temperature-dependent UV absorbance was measured using a Specord 50 Plus UV–Vis spectrophotometer (Analytik Jena) equipped with a Peltier temperature-controlled cell holder. Samples were placed in quartz cuvettes of 1 or 10 mm path length and scanned over the temperature range 20–100°C at a heating rate of 0.5°C min^−1^. Absorbance at 260 nm was recorded with a 20 s integration time. UV melting profiles were measured at DNA strand concentrations of 2 and 20 µ*M* and the melting curves were normalized. The melting temperatures (*T*_m_) for transitions were obtained from the first derivative of the optical melting curve using the *OriginPro* 7.0 software.

### 2.3. Crystallization and diffraction data collection (Tables 1 ▸ and 2 ▸)   

Crystals of all three variants were prepared using the hanging-drop vapor-diffusion method. The Hpar-18 oligo­nucleotide was crystallized using formulation G9 from the Natrix crystallization screen (Hampton Research) consisting of 30% (±)-2-methyl-2,4-pentanediol, 0.04 *M* sodium cacodylate trihydrate pH 7.0, 0.04 *M* NaCl, 0.08 *M* SrCl_2_·0.6H_2_O, 0.012 *M* spermine tetrahydrochloride. The Chom-18 and Chom-18Br oligonucleotides were crystallized in formulation G7 consisting of 22% (±)-2-methyl-2,4-pentanediol, 0.04 *M* sodium cacodylate trihydrate pH 7.0, 0.04 *M* MgCl_2_·H_2_O, 0.08 *M* SrCl_2_·0.6H_2_O, 0.012 *M* spermine tetrahydrochloride. The DNA variants crystallized within 2–5 d. The crystals did not require cryoprotection prior to flash-cooling in liquid nitrogen. A full description of the crystallization setup is given in Table 1 ▸.

The initial diffraction data were collected using a D8 Venture (Bruker) diffractometer at the Center of Molecular Structure, Institute of Biotechnology of the Czech Academy of Sciences. The final diffraction data were collected on BL14.2 at the BESSY II electron-storage ring operated by the Helmholtz-Zentrum Berlin (HZB; Mueller *et al.*, 2015 ▸). The data were processed and scaled using *XDS* (Kabsch, 2010 ▸) and *AIMLESS* (Evans & Murshudov, 2013 ▸). Diffraction measurements for the Chom-18Br variant were optimized for multiwavelength anomalous diffraction. *AIMLESS* indicated anisotropic diffraction, which was not apparent from visual inspection of the diffraction images. Significant anisotropy was observed for all three data sets. The data were further analyzed using the *STARANISO* server (Tickle *et al.*, 2018 ▸). Because the weak diffraction appeared in the *hk* plane and diffraction was strong along the *l* axis, attempts to process the data anisotropically resulted in very low data completeness (lower than 40% in a significant part of the resolution range). Therefore, a standard approach to estimate the lower resolution limit was applied. The data statistics are shown in Table 2 ▸.

### 2.4. Structure determination and refinement (Tables 2 ▸ and 3 ▸)   

The phase problem was solved using the anomalous data from Chom-18Br. Although the data were collected at four different wavelengths, phasing was only successful with the peak data (λ = 0.919831 Å) using *AutoSol* from the *Phenix* program package (Liebschner *et al.*, 2019 ▸). The presence of other heavy elements, including strontium, in the crystal structure was not anticipated and the measurements were not optimized towards their identification. Although part of the model was built automatically, extensive manual rebuilding with *Coot* (Emsley *et al.*, 2010 ▸) was necessary. Refinement was carried out with *phenix.refine* (Afonine *et al.*, 2012 ▸). The structure-refinement statistics are shown in Table 3 ▸.

Refinement was initially performed using 95% of reflections as the work set and was monitored using 5% of test (free) reflections. No water molecules were built at the given experimental resolution. The final refinement cycles were performed using all measured reflections. The valence geometry of the structures was validated by *MolProbity* (Chen *et al.*, 2010 ▸) and their conformations were validated by the tools provided by the *DNATCO* web server (https://dnatco.datmos.org/; Černý *et al.*, 2016 ▸). The tools available on this web server were also used to monitor the progress of refinement by checking the closeness of the refined geometry to the closest dinucleotide conformational (NtC) class (Schneider *et al.*, 2018 ▸; Černý *et al.*, 2016 ▸). The most probable combination of consecutive NtC classes within each structure was considered by analyzing the plots available on the *DNATCO* web server (https://dnatco.datmos.org/) under the SIMILAR tab.

The coordinates and structure factors have been deposited in the PDB with accession codes 6ror for the Chom-18Br variant, 6ros for native Chom-18 and 6rou for Hpar-18. The raw diffraction images have been deposited in Zenodo (PDB entry 6ror, https://doi.org/10.5281/zenodo.2531566; PDB entry 6ros, https://doi.org/10.5281/zenodo.2616594; PDB entry 6rou, https://doi.org/10.5281/zenodo.2616467).

## 3. Results and discussion   

The analyzed DNA oligonucleotides may theoretically exist in several structures: they can form monomeric hairpins with a canonically paired stem and a loop of unpaired TT sequence, a dimeric duplex with two T–T base pairs in the middle and also several topologies of dimeric guanine tetraplexes (Fig. 1 ▸). The theoretically possible tetramolecular quadruplexes are un­likely because mass-spectrometry data (not shown) showed no evidence for tetramolecular species in solution. Indeed, our spectroscopic measurements taken under various solution conditions indicate temperature-dependent equilibria of multiple conformational species, including both tetraplex and duplex architectures. The crystal phase revealed mismatched DNA duplexes.

### 3.1. Conformational analysis of the oligonucleotides in solution   

**Figure 2:**
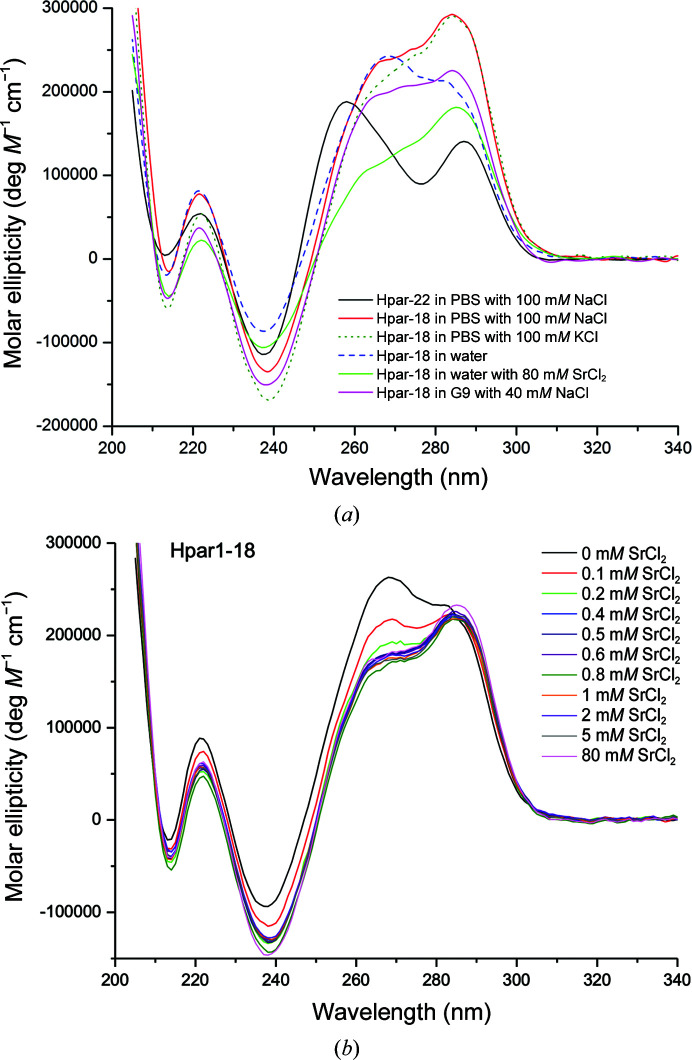
CD spectra of the Hpar-18 oligonucleotide, indicating the presence of G-­quadruplex structures in equilibrium with other conformational species. (*a*) Hpar-18 at a concentration of 20 µ*M* in various solutions compared with Hpar-22, a 22-mer consisting of the Hpar-18 sequence preceded by the RAYT-recognizing tetranucleotide GTAG. Hpar-22 data are from Charnavets *et al.* (2015 ▸). (*b*) Hpar-18 in PBS with various concentrations of Sr^2+^.

The CD spectra of all three analyzed oligonucleotides in various buffers show spectral features that are suggestive of mixtures of right-handed duplexes (Figs. 1 ▸ *a* and 1 ▸ *b*) and antiparallel G-tetraplexes (Figs. 1 ▸ *c*–1 ▸ *f*). As an example, the CD spectra of the Hpar-18 oligonucleotide in various buffers show a positive peak at 289 nm, a positive saddle at 272 nm and a negative peak at 238 nm (Fig. 2 ▸ *a*), all features that are characteristic of an antiparallel G-quadruplex architecture. The spectra have the same character as the spectrum of an oligonucleotide with the Hpar-18 sequence preceded by the RAYT-recognizing GTAG tetranucleotide (Nunvar *et al.*, 2010 ▸) at the 5′-end; this 22-mer is labeled Hpar-22 in Fig. 2 ▸(*a*). Similarly, Chom-18 and its parent GTAG-containing Chom-22 oligo­nucleotides have spectral features that are characteristic of the G-tetraplex (Supplementary Fig. S1*a*). However, as discussed in greater detail in our previous work (Charnavets *et al.*, 2015 ▸), such spectral features are not fully compatible with the CD spectra of pure ‘classic’ intramolecular antiparallel tetraplexes. The CD spectrum of a folded unimolecular or bimolecular antiparallel quadruplex would display a positive peak near 295 nm, which is often accompanied by a strong negative peak near 265 nm. This indication that the quadruplex is not the only species in solution was confirmed by an SVD analysis of the temperature-dependent CD spectra in several buffers, which revealed three to four species in a dynamic equilibrium. The absence of isodichroic points in the titration CD spectra also indicates the existence of more than two structural species in the equilibrium. Both the Hpar-18 and Chom-18 oligonucleotides exhibit a sigmoidal cooperative temperature transition at high melting temperatures, suggesting that G-tracts contribute to the stability of the folded conformation (Supplementary Figs. S2 and S3). Fig. 2 ▸(*a*) shows that the CD spectra of Hpar-18 are very similar in solutions containing only Na^+^ or phosphate-buffered saline (PBS) with added 100 m*M* K^+^. The addition of K^+^, a metal that strongly supports quadruplex formation, does not change the proportions of the molecular species. The addition of SrCl_2_ to the oligonucleotide solution also does not change the spectrum (red and green curves in Fig. 2 ▸ *a*).

The presence of species other than quadruplexes was also confirmed by measured concentration-dependent UV melting curves, which show lower melting temperatures at low oligonucleotide concentrations and higher melting temperatures at higher concentrations, which is in agreement with the previous observation by Breslauer (1995 ▸).

#### 3.1.1. The effect of strontium concentration on solution equilibria   

Because the crystallization condition contained SrCl_2_ salt, and the crystal structures contain Sr^2+^ cations, we decided to investigate how Sr^2+^ cations influence the conformation dynamics of the Hpar-18 and Chom-18 oligonucleotides in solution. We monitored the CD spectra of both 18-mers in the presence of Sr^2+^ at different concentrations. The spectra of Chom-18 and Hpar-18 are similar; Fig. 2 ▸(*b*) shows the data for Hpar-18. In pure water, both 18-mers exhibit a strong positive peak at 268 nm and a weaker peak at 283 nm. The positive peak around 270 nm is considered to be a signature of B-form duplex DNA, but can also originate from a stem of the hairpin. The positive peak at ∼285 nm can be assigned to an antiparallel quadruplex species. On successive increments in Sr^2+^ concentration, the intensity of the 268 nm duplex band decreases, while the intensity of the peak at ∼285 nm changes a little (Fig. 1 ▸ *b* and Supplementary Fig. 1*b* for Chom-18). Both these changes occur in a narrow interval of Sr^2+^ concentrations between 0.0 and 0.2 m*M*, beyond which the spectra are almost invariable even for relatively high Sr^2+^ concentrations of up to 80 m*M*.

The observed spectral transition that is induced by adding the metal cation to aqueous solution may be explained by a transformation of the duplex and/or hairpin conformations adopted in pure water to other structural species such as bimolecular tetraplexes. These experiments provided additional evidence of conformational variability of the Hpar-18 and Chom-18 oligonucleotides in solution. Similar spectra indicating dynamic equilibria of conformational species have been observed for many other sequentially related oligo­nucleotides that we have tested (data not shown). As shown in the solved crystal structures, the duplex conformation is apparently preferred in the crystal phase despite the high concentration of Sr^2+^. However, the appearance of duplexes in crystals may or may not indicate that they are the dominant conformation in solution, as crystallization is a conformation-specific process. In any case, thymine residues play an important role in the topologies outlined in Fig. 1 ▸: they either form loops of the hairpin and tetraplexes or the mismatches in the duplex.

### 3.2. The crystal structures of Chom-18, Chom-18Br and Hpar-18   

**Figure 3:**
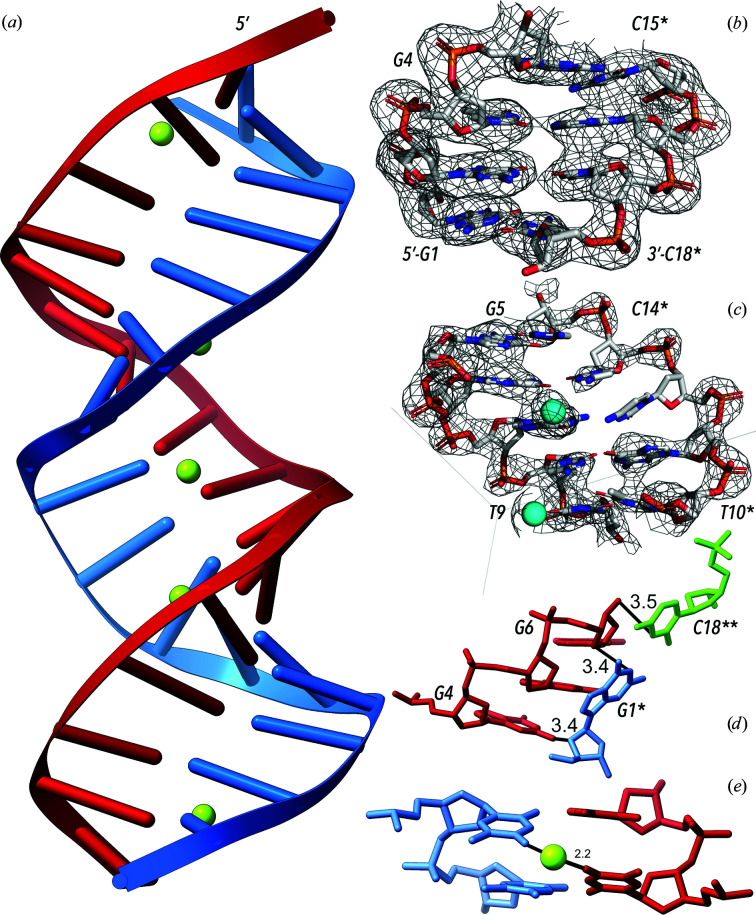
DNA 18-mer structures with two central T–T noncanonical (mismatched) base pairs. (*a*) The duplex of Chom-18 (PDB entry 6ros) with DNA strands in blue and red and Sr^2+^ cations in green. (*b*) DNA region G1–G4 and the complementary region C15–C18 from a symmetry-related chain. The anomalous Fourier map is shown as a gray mesh and is contoured at the 1.5σ level for Chom-18Br (PDB entry 6ror). (*c*) DNA region G5–T9 and the complementary region T10–C14 with poor observed (2*mF*_o_ − *DF*_c_) electron density contoured at the 1.0σ level for Chom-18 (PDB entry 6ros). (*d*) The packing of duplexes in PDB entries 6ror, 6ros and 6rou. All contacts shorter than 3.6 Å between the asymmetric unit strand (red) and the symmetry-related duplex (G1*, blue; C18**, green) are shown. (*e*) The central T–T mismatches. Sr^2+^ at the twofold axis binds to T9 and the symmetry-related T9*, as shown for Chom-18 (PDB entry 6ros).

The crystal structures of all three oligonucleotides, Chom-18, Chom-18Br and Hpar-18, were determined using highly anisotropic data at a relatively low resolution of worse than 2.6 Å. Experimental phasing was necessary because no molecular model was available. The subsequent refinement unequivocally established that all three 18-mers form antiparallel double helices in the crystal phase. The duplexes are isomorphic A-form duplexes (Fig. 3 ▸ *a*). The structures are highly similar: the calculated r.m.s.d. between all 365 non-H atoms of Chom-18 and Hpar-18 is 1.0 Å and the r.m.s.d. between Chom-18 and Chom-18Br is 0.24 Å. The asymmetric units contain single DNA strands; the biological unit, the DNA duplex, is generated by the crystallographic twofold symmetry axis. The duplexes are composed of two segments formed by eight canonical Watson–Crick base pairs divided by two noncanonical T–T pairs. The three reported structures are among the longest DNA duplexes in the database. A B-like duplex built of Watson–Crick pairs (PDB entry 5f9i; S. Garcia, F. J. Acosta-Reyes, N. Saperas & J. L. Campos, unpublished work) is a 20-mer and the structures in PDB entries 5vy6 and 5vy7 are self-assembling duplexes composed of four strands, one of which has a length of 21 nucleotides (Simmons *et al.*, 2017 ▸).

The crystal structures contain one central and one (in Hpar-18 and Chom-18Br) or two (in Chom-18) peripheral Sr^2+^ cations. The central Sr^2+^ cation is located on the twofold symmetry axis generating the duplex, and binds to two symmetry-related major-groove O4 atoms of T9 (Fig. 3 ▸ *e*). The distance between thymine O4 and Sr^2+^ in all three crystal structures is between 2.2 and 2.4 Å. The peripheral Sr^2+^ cations were refined with partial occupancy and bind loosely to just one of the strands. Because of the limited resolution, no water molecules were observed in any of the presented structures. In all cases the crystallization solutions contained Na^+^, a quadruplex-inducing metal, but also the quadruplex-breaking Mg^2+^ (in Chom-18 and Chom-18Br) and Li^+^ (in Hpar-18). As all three solutions share Sr^2+^, which is also observed in crystallographically defined positions, we conclude that the strontium cation was essential for successful crystallization.

The Protein Data Bank contains 24 DNA crystal structures that contain Sr^2+^ cations. The metals are involved in a number of interactions, for example in water-coordinated binding to a DNA duplex (PDB entry 3v06; Pallan *et al.*, 2012 ▸), as several Sr^2+^ cations coordinated to the bases as well as the phosphates of an DNA duplex (PDB entry 1wv6; Egli *et al.*, 2005 ▸), involved in outer-shell binding to phosphates in a Holliday junction structure (PDB entry 1m6g; Thorpe *et al.*, 2003 ▸) and participating in the crystal packing of a telomeric DNA segment containing a quadruplex motif (PDB entry 6h5r; Guarra *et al.*, 2018 ▸). The crystal structures of A-like duplexes d(GGTCGT­CC)_2_ (PDB entries 5wsp and 5gsk; Liu *et al.*, 2017 ▸) show the same binding of Sr^2+^ to the symmetry-related mismatched thymines, O4(T)⋯Sr^2+^⋯O4(T)*, as we observe in the reported structures. Also in analogy to our structures, both steps involved in the T–T mismatch in PDB entries 5wsp and 5gsk are classified as typical A-form NtC classes AA00 (G2T3) and AA08 (T3C3) and do not therefore deform the regular duplex architecture.

#### 3.2.1. Crystal packing   

In all three reported structures, the duplex is formed by a twofold axis dissecting the T–T mismatches. The packing of duplexes is mediated by contacts between nucleotides G4 and G6 of one strand and the symmetry-related pair G1*–C18** of another duplex (Fig. 3 ▸ *d*). The deoxyribose ring of G6 stacks on the symmetry-related base pair G1*–C18**, and the deoxyribose O4′ atom of G1* intrudes into the minor groove of G4, forming a weak N2–O4′* hydrogen bond (3.4 Å in length). This packing mode is reminiscent of the packing observed in octamers such as d(GGGGCCCC)_2_ (PDB entry 2ana; McCall *et al.*, 1985 ▸) and decamers, for example d(GCGGGCCCGC)_2_ (PDB entries 137d and 138d; Ramakrishnan & Sundaralingam, 1993 ▸), where two neighboring sugar rings of one strand stack on the first pair of a symmetry-related duplex. In all three cases, the hydrophobic surfaces of the terminal base pairs stack on the sugar ring edges and may form a few direct or water-bridged (PDB entries 136d and 137d) hydrogen bonds. It is notable that these similar packing interactions occur for duplexes of different lengths of 18, ten and eight nucleotides. All of these duplexes are arranged in a zigzag pattern, but they are arranged in crystal lattices belonging to different space groups.

It is known that the packing influences or in some cases perhaps directly induces the A-form in the crystal form (reviewed in Wahl & Sundaralingam, 1997 ▸), but the general preference of sequences such as dG_*n*_·dC_*n*_ to form the A-form, especially in high-salt solutions, is known from solution studies. The A-form is by no means a crystallization artifact and plays an important role in protein–DNA recognition. Deformations of the duplex to the local A-form are prototypical in TATA box-binding transcription factors, as documented by many structures, for example PDB entries 1ytb (Kim *et al.*, 1993 ▸) and 4roc (Gouge *et al.*, 2015 ▸). DNA bending by a locally induced A-form is typical in the binding of DNA by many transcription factors. In contrast, the wrapping of DNA around the histone core particle is achieved by the periodic transition of the prevailing BI to BII or related conformers: transition between BB00 and BB07 in the NtC nomenclature (Schneider *et al.*, 2017 ▸).

### 3.3. The Chom-18, Chom-18Br and Hpar-18 structures annotated with help of the dinucleotide conformational (NtC) classes   

The dinucleotide conformational (NtC) classes (Schneider *et al.*, 2018 ▸; Černý, Božíková, Svoboda *et al.*, 2020 ▸) allow the objective classification of DNA and RNA geometries. The classification is automated and is available at the web site https://dnatco.datmos.org/ (Černý *et al.*, 2016 ▸), where DNA- or RNA-containing structures in mmCIF or PDB format are dissected into dinucleotide blocks that are then assigned to NtC classes, with a related goodness-of-fit measure (confal) and several other characteristics. The web service also measures how well the dinucleotide fragments fit into electron density (when available). The 96 NtCs describe the local geometry of DNA or RNA; one class is reserved for geometrically unassigned dinucleotides. The NtC classes are grouped into the 15 codes of the CANA (Conformational Alphabet of Nucleic Acids) structural alphabet that enables a symbolic annotation of the prominent structural features of nucleic acids. Here, we use the NtC and CANA classifications to annotate the newly solved structures with PDB codes 6ror, 6ros and 6rou and discuss their structural features; the results of the assignment are summarized in Supplementary Table S1.

The A-like character of all three duplexes is confirmed by the dominance of NtC classes describing the A form, with the ‘canonical’ AA00 and the common AA08 prevailing. The structures also contain the less frequent NtC classes AA06, AA10 and AA11 that have unusual combinations of torsions α and γ plus low or high values of torsion β, but are fully compatible with the regular A-DNA duplex. In both the Chom-18 and Chom-18Br structures, all but two central steps (10–11–12) are assigned to NtC classes, while in Hpar-18 two additional steps, 4–5 and 12–13, cannot be assigned and are formally assigned NtC class NANT. However, the unassigned steps are conformationally close to the A-like NtC classes, with a small r.m.s.d. from the closest NtC representatives of lower than 0.6 Å. A-like NtC classes are also assigned to the dinucleotides with T–T mismatches, as discussed below.

#### 3.3.1. Improvement of the fit to the electron density   

In the reported structures, all nucleotides have been identified in the observed electron density. While density for nucleotides from G1 to C8 and from C14 to C18 was highly visible, the quality of the electron density between nucleotides T9 and C13 was limited and the region T10-G11-C12 was only visible as a low-resolution blob. Surprisingly, the electron density for nucleotides from the strand opposite T10-G11-C12 was well defined. Model building in this region would be very difficult without experimental phases (Figs. 3 ▸ *b* and 3 ▸ *c*) and detailed knowledge of the geometries of the NtC classes and the analytical functions available at the *DNATCO* web server significantly helped to improve the fit of the refined models to the experimental data.

The observed electron density in the T10-G11-C12 region was not of sufficient quality to guide manual model building. This fact was reflected by a poor overlap between the manually fitted geometries of the dinucleotides T10-G11 and G11-C12 and the geometry of any known NtC class (Schneider *et al.*, 2018 ▸; Černý, Božíková, Svoboda *et al.*, 2020 ▸). An in-depth geometric analysis of these dinucleotides in the pre-final coordinates indicated the possibility of improving their geometric fit to the target NtC geometries. This improvement of the geometric fit was carried out by an iterative manual process involving gradual geometry changes directed by calculations at https://dnatco.datmos.org.

The process led to a decrease in the *R*_work_ and *R*_free_ values, but the rebuilding of structural models with help from the NtC geometries was laborious and was fully dependent on manual intervention. The above-described improvement of the DNA fragment in low-density regions between T10 and C12 needs to be replaced by an automated, program-driven procedure. To test the parameters for a procedure that will be able to refit the geometries to comply closer with the known NtC classes, we inspected the PDB-deposited structures with *PDB_REDO* (Joosten *et al.*, 2014 ▸). The dinucleotides with unclassified geometries (NtC class NANT) showed an improved agreement between the re-refined geometries and the geometries of the closest NtC class in cases when the r.m.s.d. between the initial PDB-deposited geometry and the NtC target was smaller than 1 Å (the r.m.s.d. was measured for 18 atoms which define the NtC geometry). R.m.s.d. values of larger than 1 Å typically indicate geometry deviations that are too large to be remediated by the current algorithms implemented in *PDB_REDO*.

Our experience with building a molecular model into relatively low-resolution and featureless electron density points to the need to develop more powerful refinement protocols that would simultaneously respect both the experimental electron density and predetermined target geometries (such as the NtC classes in our case). Therefore, we propose the implementation and application of NtC restraints in the refinement of nucleic acids as a tool for the overall improvement of the quality of the geometry of a model. This task seems timely, especially in the light of emerging low-resolution cryo-EM structures.

### 3.4. The geometry of T–T mismatches   

#### 3.4.1. T–T mismatches in the reported structures   

The central region of the studied Chom-18 and Hpar-18 duplexes contains two consecutive T–T mismatches (Fig. 3 ▸ *e*). Both thymine pairs can be classified as number 1 according to the Leontis–Westhof nomenclature (Leontis & Westhof, 2001 ▸) and number 16 according to the Saenger nomenclature (Saenger, 1984 ▸). In all three structures the central Sr^2+^ links the two O4 atoms of the symmetry-related mismatched thymines T9–T9*. The crystallographically unique TT di­nucleotides forming the mismatches, residues T9 and T10, are assigned to the frequently occurring NtC class AA08 in all three structures, with the preceding C8-T9 assigned to AA08 or AA00 and the following T10-G11 unassigned (NtC NANT). Therefore, the mismatched base pairs do not necessarily deform the sugar-phosphate backbone into a ‘unique’ unclassifiable conformation. The backbone deformation to the unclassifiable NANT conformation is asymmetrically shifted in the 3′ direction of the DNA strand. It still needs to be tested whether this is a more general feature of duplexes with mismatched pairs or whether it is just a coincidental detail of the reported structures.

#### 3.4.2. T–T mismatches in PDB-deposited structures   

T–T mismatches were found in 27 crystal structures containing DNA (six of naked DNA and 21 protein–DNA complexes; a list of the PDB codes is given in the supporting infomation and was obtained from the PDB release of 5 November 2019) that contain 45 incidences of T–T mismatches. Three found in parallel strand structures were assigned to the Saenger pairing class 12; the remaining 42 in the antiparallel duplexes are all Saenger class 16. Dinucleotides containing T–T mismatches are assigned to the NtCs NANT (about a quarter), BB00 and AA00 (each just below a fifth); all other NtCs account for less than 40%. No structure other than the three reported here contains two successive T–T mismatches. On the other hand, sequentially subsequent U–U mismatches are known in RNA double helices, for instance in PDB entry 205d (Baeyens *et al.*, 1995 ▸), where the dinucleotide U6-U7 is mispaired with the slightly unstacked U18-U19 (NtC AA12). Similarly to our structures, the mismatched region does not deform the A-like duplex.

#### 3.4.3. An attempt to analyze the geometries of all mismatched base pairs   

The presence of two successive non­canonical T–T pairs in our structures prompted a more systematic analysis of noncanonical pairs in the deposited structures. We searched the mmCIF token ndb_struct_na_base_pair.hbond_type_28 for values other than ‘19’, ‘20’ or ‘?’ denoting the canonical Watson–Crick or unknown pairing types, respectively, and retrieved 1094 base-paired dinucleotides with at least one pair in a noncanonical arrangement (4447 structures with resolution better than 3.0 Å in the PDB release of 5 November 2019).

The incidences of noncanonical pairs are listed in Table 4 ▸ separately for the parallel and antiparallel strands. The most populated noncanonical pairs are A–G, A–T, C–G and G–T. Some mismatched base pairing was found only in antiparallel strands (A–G, G–T, C–G, C–T and A–C); on the other hand, C–C base pairs were only found in parallel strands in i-motif structures. C–G and A–T can form noncanonical pairs, but their high incidence in the DNA structures indicated by the mmCIF category ndb_struct_na_base_pair.hbond_type_28 is indeed surprising. We randomly checked about 50 of these supposedly noncanonical pairs and found that the majority were misclassified: while they were classified as non­canonical, they formed Watson–Crick pairs.

#### 3.4.4. The geometry and fit to electron density of dinucleotides containing noncanonical pair(s)   

Despite the classification of base pairing in the mmCIF archival files needing a thorough revision, we decided to analyze the pool of retrieved dinucleotides (Table 4 ▸). Firstly, we calculated how close their geometries are to the geometry of the closest NtC class. The fit was calculated as the root-mean-square deviation (r.m.s.d.) between the investigated dinucleotide and the geometrically closest dinucleotide from the ensemble of dinucleotides defining the NtC classes (Černý, Božíková, Svoboda *et al.*, 2020 ▸). In the following step, we measured the real-space correlation coefficient (RSCC; Authier & Chapuis, 2014 ▸) for the investigated mismatched dinucleotides. RSCC was calculated using *phenix.real_space_correlation* (Adams *et al.*, 2010 ▸). Both the RSCC and r.m.s.d. were calculated for the 18 atoms that define the dinucleotide geometry (Černý, Božíková, Malý *et al.*, 2020 ▸). The scattergrams of the RSCC versus r.m.s.d. values represent a new type of correlative analysis that allows the identification of fragments that are in (dis)agreement with the known conformation and experimental electron density.

**Figure 4:**
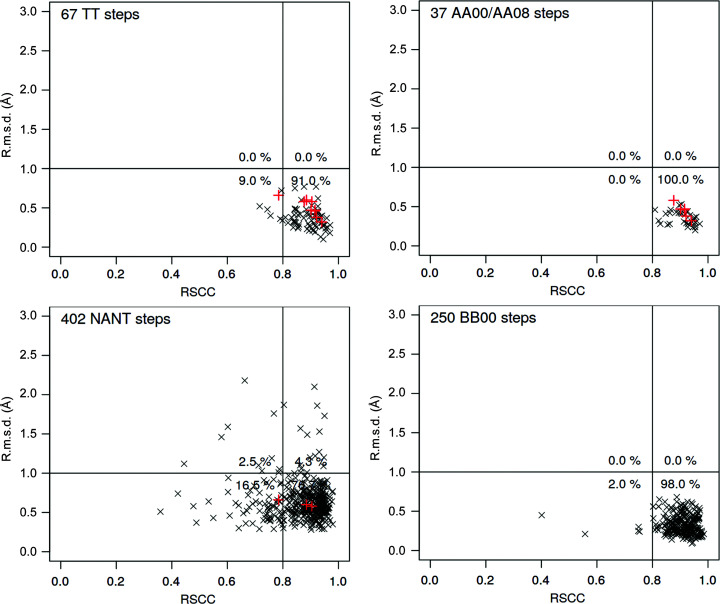
Scattergrams showing the relationship between the fit to electron density (measured as the real-space correlation coefficient; RSCC) and the geometric fit between the dinucleotide geometry and the geometrically closest dinucleotide in the ‘golden set’, an ensemble of dinucleotides defining the NtC classes (r.m.s.d.) (Černý, Božíková, Svoboda *et al.*, 2020 ▸). The data were calculated for dinucleotides containing at least one base forming a noncanonical base pair. The top left scattergram reports on dinucleotides with the T–T mismatches and the other three on dinucleotides with mismatches as listed in Table 4 ▸. The red crosses highlight data from the three reported structures: PDB entries 6ror, 6ros and 6rou. The RSCC–rm.s.d. and analogus scattergrams were calculated for all dinucleotides in the archives classified into all 96 + 1 NtC classes. They can be seen at https://dnatco.datmos.org/contours .

Fig. 4 ▸ shows four such correlations, one for dinucleotides containing T–T mismatches and three for the dinucleotides with any mismatch and classified as AA00 or AA08, BB00 or not classified (NANT), respectively. In all graphs, values for the reported structures are highlighted in red. Data points in the lower right rectangle of each graph show dinucleotides that fit well into electron density and with geometries close to the geometries of the known NtC classes. This is true even for the unassigned dinucleotides because their geometries are also compared with the geometries of well defined conformers. These geometries can be close even for the NANT dinucleotides because the r.m.s.d.s are calculated in Cartesian coordinates but the NtC assignment is a complex algorithm performed in torsion space. The scattergrams in Fig. 4 ▸ show that a majority of the mismatched dinucleotides are classified as known and are actually the most common conformers AA00, AA08 and BB00, and also other common NtC classes such as BB01 and the mixed A/B conformers BA05 and AB01, for which the scattergrams are not shown (the RSCC–r.m.s.d. and other scattergrams for all 96 + 1 NtC classes can be seen at https://dnatco.datmos.org under ‘About’). Even more important is the fact that the majority (three quarters) of unclassified dinucleotides (NtC class NANT) fit well into electron density while their geometry is simultaneouly close to a known NtC class. This means that they are likely to become compliant with the known conformers upon a re-refinement process using properly defined restraints. To conclude, we do not observe major deformations of the backbone geometry caused by the mispairing.

## 4. Conclusions   

We studied a specific class of bacterial noncoding single-stranded DNA segments called repetitive extragenic palindromes (REPs). The biologically relevant form of REPs is considered to be a hairpin with the GTAG recognition tetranucleotide, a right-handed stem linked by a short turn (Messing *et al.*, 2012 ▸). In this work, we studied several REP-related oligomers, emphasizing the results obtained for two 18-mers from two bacterial species. Solution studies using CD and UV spectroscopy (Fig. 2 ▸ and Supplementary Figs. S1–S3) confirmed the results of our previous study (Charnavets *et al.*, 2015 ▸) showing that CG-rich, near-palindromic REPs can adopt structures other than hairpins. The results indicate dynamic equilibria between the right-handed form(s) and tetraplex architectures formed by two or four strands; the possible topologies are outlined in Fig. 1 ▸. All topologies stress the importance of thymine residues: they either form loops of the hairpin and tetraplexes or the mismatches in the duplex.

Crystallization attempts were successful for three of the studied REP-related 18-mers named Hpar-18 (PDB entry 6rou), Chom-18 (PDB entry 6ros) and the brominated variant Chom-18Br (PDB entry 6ror). The crystals produced anisotropic and relatively low-resolution diffraction (Tables 1 ▸, 2 ▸ and 3 ▸) that was phased using the bromine anomalous signal of Chom-18Br. All three structures revealed an asymmetric unit composed of one 18-mer strand that formed a right-handed A-like duplex by applying a twofold-symmetry operation (Fig. 3 ▸). The center of the duplex is formed by two successive T–T mismatches. Detailed structural analysis of the structures was performed by assigning the dinucleotide conformer (NtC) classes (Schneider *et al.*, 2018 ▸; Černý, Božíková, Svoboda *et al.*, 2020 ▸) to their dinucleotides using the *DNATCO* web server (https://dnatco.datmos.org; Černý *et al.*, 2016 ▸; Černý, Božíková, Malý *et al.*, 2020 ▸). The assignment revealed a majority of A-like NtC classes; a detailed assignment is given in Supplementary Table S1.

Our experience with building a molecular model into relatively low-resolution and featureless electron density around the dinucleotide T10-G11 points to the need to develop more powerful refinement protocols that would respect both experimental electron density and predetermined target geometries such as NtC classes, and we propose the implementation of restraints based on the NtC geometries in refinement protocols. The ascent of cryo-electron microscopy, providing an increased number of low-resolution structures, provides further demand for this task.

In all three crystals, the T9-T10 mismatched dinucleotides acquire the geometry assigned to the AA08 class, which is the second most common A-form conformer. We therefore performed an analysis of DNA dinucleotides containing T–T and other mismatches across the database. This revealed that their geometries also adopt similar conformations to di­nucleotides involved in Watson–Crick pairs (Fig. 4 ▸) and that the mispaired nucleotides do not impose major deformations of the backbone geometry. Unfortunately, we found serious inconsistencies in the information about pairing in the archival mmCIF files, where many A–T and C–G pairs are incorrectly labeled as noncanonical (Table 4 ▸). The base-pairing information of DNA and RNA structures requires revision.

This analysis of REP-related 18-mer DNA oligonucleotides demonstrates the complexity of DNA conformational space. Our understanding of DNA dynamic equilibria and their role in biology is still limited and requires a combination of experimental techniques and likely novel approaches for their analysis. Here, we show one possible direction by applying the automated geometric classification of dinucleotide fragments using the NtC classes (Schneider *et al.*, 2018 ▸; Černý, Božíková, Svoboda *et al.*, 2020 ▸).

## Supplementary Material

PDB reference: Chom-18, 6ros

PDB reference: Chom-18Br, 6ror

PDB reference: Hpar-18, 6rou

Supplementary Figures and Tables. DOI: 10.1107/S2059798320014151/cb5122sup1.pdf

REP related 18-mer DNA (Chom22Br) - diffraction data: https://doi.org/10.5281/zenodo.2531566

REP related 18-mer DNA (Chom22) - diffraction data: https://doi.org/10.5281/zenodo.2616594

REP related 18-mer DNA (Hpar1) - diffraction data: https://doi.org/10.5281/zenodo.2616467

## Figures and Tables

**Table 1:** Crystallization conditions

Method	Hanging-drop vapor-diffusion method
Plate type	Linbro plates (24-well)
Crystallized sequences	
Chom-18Br	GGTGGGGC(BrU)TGCCCCACC
Chom-18	GGTGGGGCTTGCCCCACC
Hpar-18	GGTGGGTCTTGACCCACC
Temperature (K)	293 (Chom-18 variants), 297 (Hpar-18)
DNA concentration	Approximately 0.5 m*M*: 1 µl 1.5 m*M* oligonucleotide stock mixed with 2 µl buffer
Composition of reservoir solution	
Chom-18 variants	22–26% (±)-2-methyl-2,4-pentanediol, 0.04 *M* sodium cacodylate trihydrate, 0.04 *M* magnesium chloride hexahydrate, 0.08 *M* strontium chloride hexahydrate, 0.012 *M* spermine tetrahydrochloride
Hpar-18	30–32% (±)-2-methyl-2,4-pentanediol, 0.04 *M* sodium cacodylate trihydrate, 0.04 *M* lithium chloride, 0.08 *M* strontium chloride hexahydrate, 0.012 *M* spermine tetrahydrochloride
Volume and ratio of drop	2 µl, 1:1
Volume of reservoir (µl)	1000

**Table 2:** Data collection and processing Values in parentheses are for the outer shell.

	Chom-18Br	Chom-18	Hpar-18
Diffraction source	MX 14.2, HZB	MX 14.2, HZB	MX 14.2, HZB
Wavelength (Å)	0.919831	0.979491	0.979491
Temperature (K)	100	100	100
Crystal-to-detector distance (mm)	326	289	304
Rotation range per image (°)	0.1	0.1	0.1
Total rotation range (°)	357	360	210
Exposure time per image (s)	0.2	0.2	0.2
Space group	*P*4_3_2_1_2	*P*4_3_2_1_2	*P*4_3_2_1_2
*a*, *b*, *c* (Å)	38.47, 38.47, 90.78	38.44, 38.44, 89.58	38.56, 38.56, 89.94
α, β, γ (°)	90, 90, 90	90, 90, 90	90, 90, 90
Mosaicity (°)	0.2	0.2	0.2
Resolution range (Å)	45.4–2.6 (2.72–2.60)	44.8–2.7 (2.85–2.70)	45.0–2.9 (3.10–2.90)
Total No. of reflections	53968 (6716)	47846 (6928)	23027 (4393)
No. of unique reflections	2401 (276)	2126 (290)	1758 (299)
Completeness (%)	100.0 (100.0)	99.9 (100.0)	99.9 (100.0)
Multiplicity	22.5 (24.3)	22.5 (23.9)	13.1 (14.7)
〈*I*/σ(*I*)〉	26.7 (1.4)	24.7 (2.2)	20.0 (3.7)
*R*_p.i.m._	0.013 (0.590)	0.014 (0.372)	0.019 (0.188)
Overall *B* factor from Wilson plot (Å^2^)	89	88	116

**Table 3:** Structure solution and refinement Values in parentheses are for the outer shell.

	Chom-18Br	Chom-18	Hpar-18
No. of reflections, working set	2259	1790	1629
No. of reflections, test set	108	95	94
Final *R*_work_	0.239	0.242	0.264
Final *R*_free_	0.292	0.279	0.312
Final *R*_all_	0.239	0.250	0.272
No. of non-H atoms			
DNA	365	365	365
Ion	2	3	2
Total	367	368	367
R.m.s. deviations			
Bonds (Å)	0.010	0.011	0.010
Angles (°)	1.21	1.09	1.02
Average *B* factors (Å^2^)	120	108	122
NtC analysis			
Assigned	15	15	14
Outliers	2	2	3
PDB code	6ror	6ros	6rou

**Table 4:** Incidences of noncanonical base pairs in parallel and antiparallel strands as retrieved from the ndb_struct_na_base_pair.hbond_type_28 mmCIF token in 4447 DNA-containing structures All base-pair combinations are listed, including A–T and C–G pairs.

Base pair	A–A	A–C	A–G	A–T	C–C	C–G	C–T	G–G	G–T	T–T
Antiparallel	16	8	175	193	0	127	14	72	141	42
Parallel	34	0	0	1	115	0	0	153	0	3
