# Supplementary material for: Structural variability of CG-rich DNA 18-mers accommodating double T–T mismatches
Source: Acta Crystallogr D Struct Biol. 2020 Nov 24;76(Pt 12):1233–43. doi: 10.1107/S2059798320014151 (PMC7709200; doi:10.1107/S2059798320014151)
Supplement: Supplementary file 1 [file d-76-01233-sup1.pdf]

STRUCTURAL BIOLOGY

Volume 76 (2020)

Supporting information for article:

Structural variability of CG-rich DNA 18-mers accommodating double T–T mismatches

Petr Kolenko, Jakub Svoboda, Jiří Černý, Tatsiana Charnavets and Bohdan Schneider

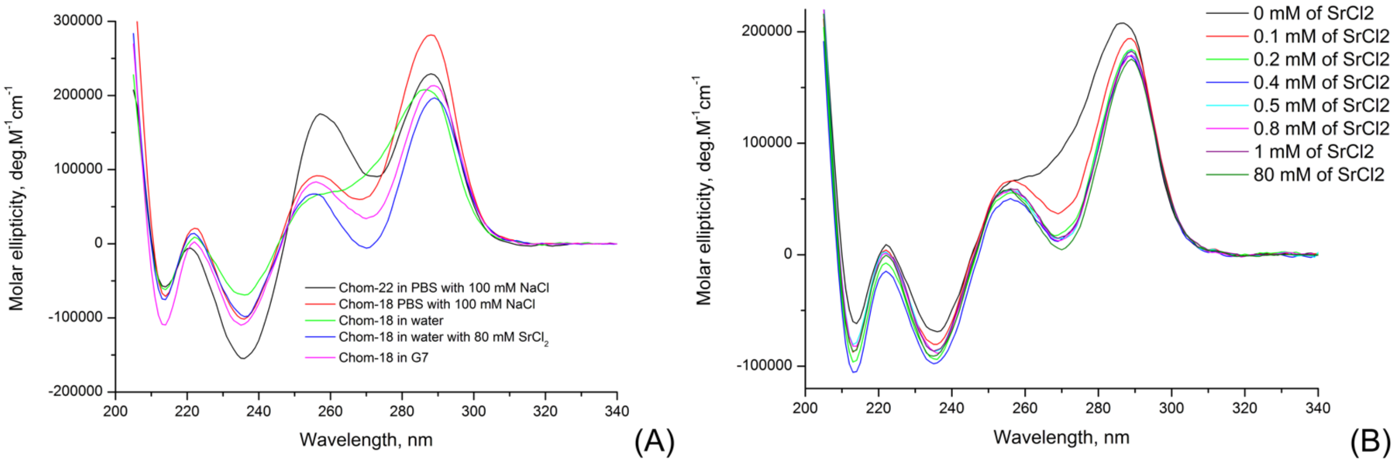

Figure S1. CD spectra of Chom oligonucleotides at concentration 20 μM in different solutions: PBS buffer with 100 mM NaCl, water, water with 80 mM $SrCl_2$ and G7 (A); on $SrCl_2$ titration (B).

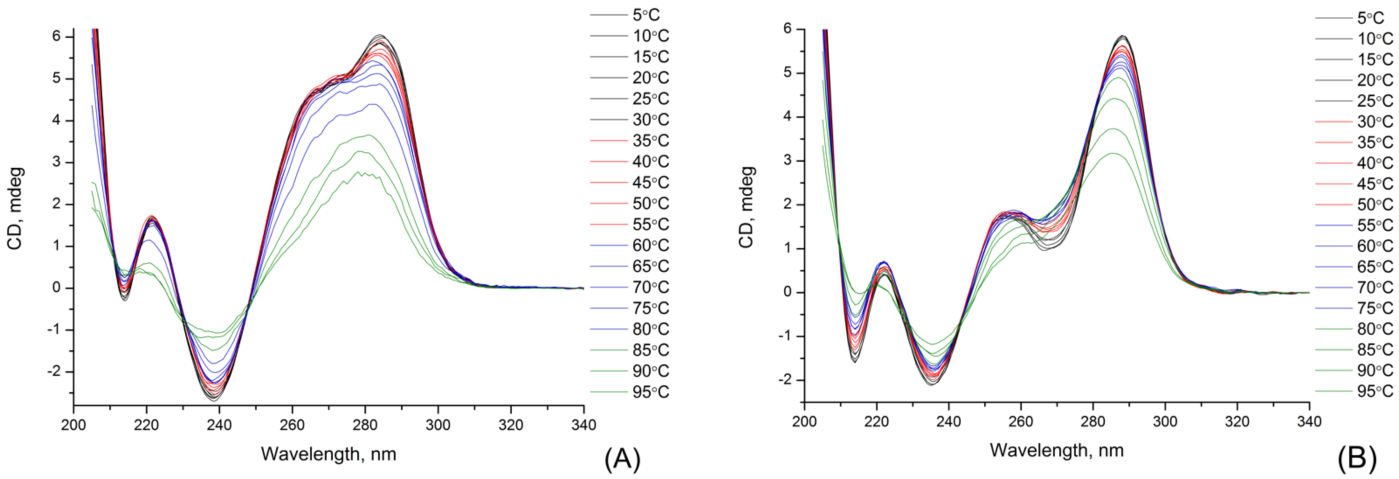

Figure S2. Temperature-dependent CD spectra of Hpar-18 (A) and Chom-18 (B) oligonucleotides at concentration 20 μM in PBS buffer with 100 mM NaCl.

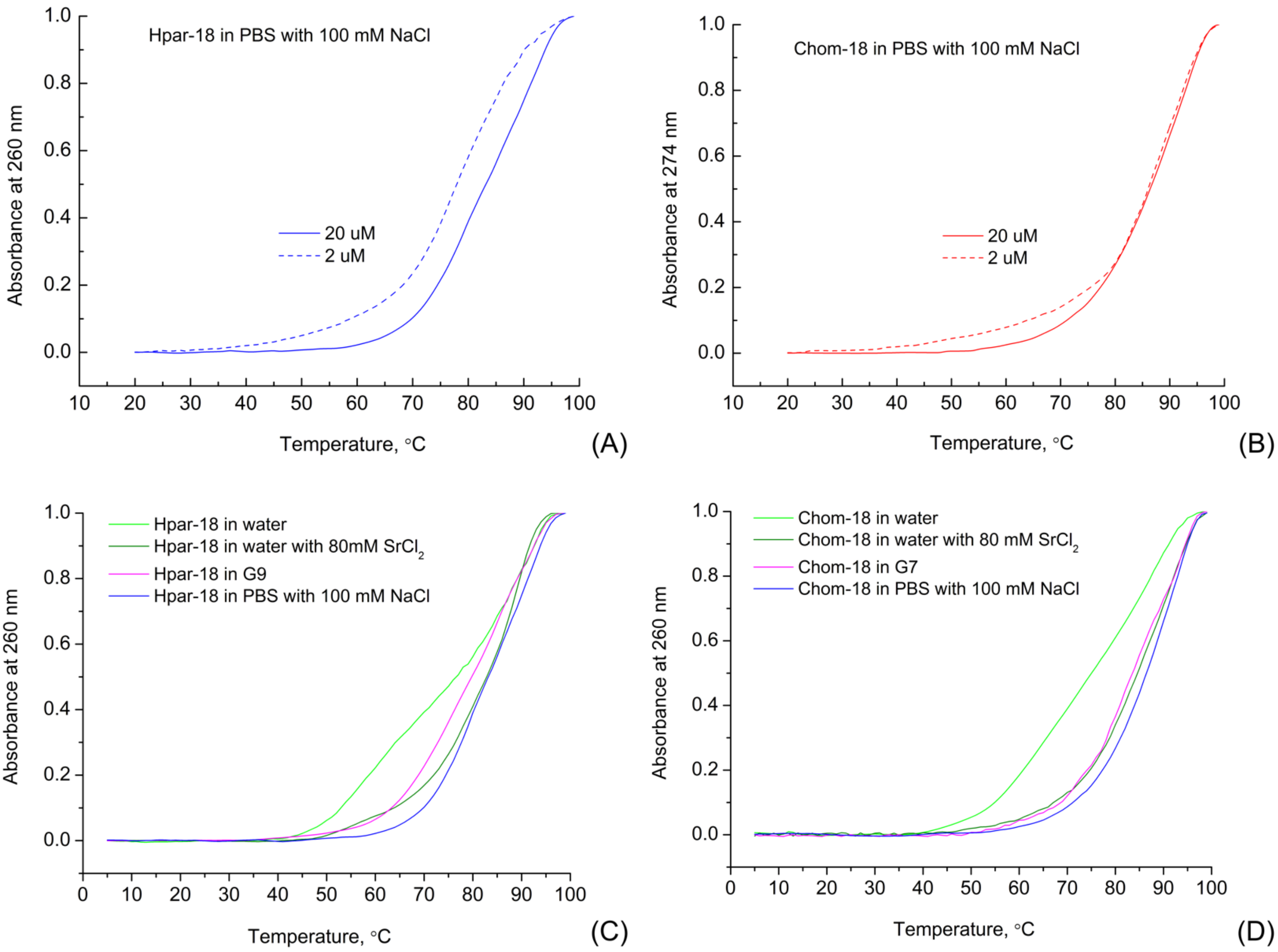

Figure S3. Normalized UV melting curves of Hpar-18 and Chom-18 oligonucleotides. Hpar-18 at concentrations 2 and 20 μM in PBS buffer with 100 mM NaCl (A), and Chom-18 under the same conditions (B). Normalized UV melting curves of Hpar-18 (C) and Chom-18 (D) oligonucleotides at concentration 20 μM in different solutions: water, water with 80 mM $SrCl_2$, screen formulations, PBS buffer with 100 mM NaCl.

Table S1. Assignment of dinucleotide classes NtC (Schneider *et al*. 2018) to the crystal structures Chom-18Br, PDB ID 6ROR, Chom-18, PDB ID 6ROS, and Hpar-18, PDB ID 6ROU.

Chom-18Br 6ROR	NtC	CANA	confal	nearest NtC	rmsd
DG_1_DG_2	AA08	AAA	75	AA08	0.26
DG_2_DT_3	AA00	AAA	83	AA00	0.16
DT_3_DG_4	AA00	AAA	68	AA00	0.25
DG_4_DG_5	AA04	AAA	59	AA04	0.32
DG_5_DG_6	AA00	AAA	72	AA00	0.20
DG_6_DG_7	AA01	AAw	58	AA01	0.21
DG_7_DC_8	AA08	AAA	52	AA08	0.32
DC_8_BRU_9	AA08	AAA	63	AA08	0.32
BRU_9_DT_10	AA08	AAA	10	AA08	0.38
DT_10_DG_11	NANT	NAN	0	AA10	0.60
DG_11_DC_12	NANT	NAN	0	BB10	0.57
DC_12_DC_13	BA08	B-A	52	BA08	0.41
DC_13_DC_14	AA00	AAA	76	AA00	0.18
DC_14_DC_15	AA08	AAA	79	AA08	0.30
DC_15_DA_16	AA06	AAw	93	AA06	0.28
DA_16_DC_17	AA08	AAA	79	AA08	0.24
DC_17_DC_18	AB05	A-B	64	AB05	0.21

Chom-18 6ROS	NtC	CANA	confal	nearest NtC	rmsd
DG_1_DG_2	AA08	AAA	61	AA08	0.30
DG_2_DT_3	AA00	AAA	68	AA00	0.24
DT_3_DG_4	AA08	AAA	87	AA08	0.16
DG_4_DG_5	AA04	AAA	87	AA04	0.23
DG_5_DG_6	AA00	AAA	74	AA00	0.17
DG_6_DG_7	AA10	AAw	75	AA10	0.18
DG_7_DC_8	AA08	AAA	72	AA08	0.24
DC_8_DT_9	AA00	AAA	57	AA00	0.46
DT_9_DT_10	AA08	AAA	20	AA08	0.47
DT_10_DG_11	NANT	NAN	0	AA10	0.58
DG_11_DC_12	NANT	NAN	0	AB03	0.55
DC_12_DC_13	BA08	B-A	41	BA08	0.43
DC_13_DC_14	AA00	AAA	71	AA00	0.17
DC_14_DC_15	AA08	AAA	84	AA08	0.26
DC_15_DA_16	AA06	AAw	88	AA06	0.27
DA_16_DC_17	AA08	AAA	81	AA08	0.24
DC_17_DC_18	AB05	A-B	63	AB05	0.23

Hpar-18 6ROU	NtC	CANA	confal	nearest NtC	rmsd
DG_1_DG_2	AA04	AAA	79	AA04	0.28
DG_2_DT_3	AA00	AAA	82	AA00	0.15
DT_3_DG_4	AA00	AAA	91	AA00	0.18
DG_4_DG_5	NANT	NAN	0	AA10	0.45
DG_5_DG_6	AA08	AAA	26	AA08	0.35
DG_6_DT_7	AA11	AAw	36	AA11	0.44
DT_7_DC_8	AA08	AAA	73	AA08	0.32
DC_8_DT_9	AA00	AAA	51	AA00	0.47
DT_9_DT_10	AA08	AAA	21	AA08	0.58
DT_10_DG_11	NANT	NAN	0	AA10	0.66
DG_11_DA_12	NANT	NAN	0	AA03	0.57
DA_12_DC_13	NANT	NAN	0	BA05	0.34
DC_13_DC_14	AA00	AAA	38	AA00	0.28
DC_14_DC_15	AA08	AAA	90	AA08	0.29
DC_15_DA_16	AA06	AAw	69	AA06	0.47
DA_16_DC_17	AA08	AAA	64	AA08	0.25
DC_17_DC_18	AA00	AAA	57	AA00	0.20

PDB IDs of structures containing T-T base pairs (PDB release of 2019-11-05): 6BOW 3DSD 4Q0W 4QJU 3WPD 3WPG 3WPH 3ZVN 3G00 3LDY 4RIM 4RIP 3M7K 2QSH 2IS4 2BCU 1MNV 1NJY 1P51 1P71 1P78 5WSP 5YTZ 5J0Y 5J2K 5LS8 4DAV

Reference

Schneider, B., Bozikova, P., Necasova, I., Cech, P., Svozil, D. & Cerny, J. (2018). Acta Cryst D74, 52-64.
